# New Directions for Preventing Dating Violence in Adolescence: The Study of Gender Models

**DOI:** 10.3389/fpsyg.2018.00946

**Published:** 2018-06-12

**Authors:** Chiara Santoro, Belén Martínez-Ferrer, Carmen Monreal Gimeno, Gonzalo Musitu

**Affiliations:** Department of Education and Social Psychology, Pablo de Olavide University, Seville, Spain

**Keywords:** dating violence, adolescence, gender models, gender policies, prevention

## Abstract

Dating violence is a huge transcultural and alarming phenomenon, directly linked with endless discrimination against women. The latest research on dating violence in adolescence shows how dating violence is persistent and common in the adolescent period as well and pinpoints the origin of gender violence from first adolescent relationships. This element takes us to considerate how recent gender violence studies and policies, increased also thanks to international efforts on this issue, are not bringing expected results, especially among young people. This mini-review aims to analyze the main characteristics of current gender studies and policies on dating violence, focusing on percentages with a woman-centered approach, which stresses the consequences of gender violence. Other gender studies, that consider gender as a relational product, stress the importance of integrating the analysis of gender models as a key instrument to understand the main causes of dating violence, providing new elements to develop effective policies against dating violence. Indeed, gender models of femininity and masculinity are based on a binary system, which is also a reciprocal recognition and identity system: gender models define female and male characteristics, roles, stereotypes, and expectation, being complementary and foreclosing at the same time. Recent studies on gender relationships, especially among the youth, allows us to propose a new dialog between dating violence studies and gender model studies, underling the need of a complete and complex understanding of gender structure, and of its tensions and contradictions, to put an end to gender and dating violence, through effective programs.

## Introduction

In the context of gender violence, dating violence is a particularly worrying phenomenon. The World Health Organization (WHO) affirms that one in three women has been a victim of physical or sexual violence by an intimate partner at some point in her lifetime and, globally, as many as 38% of all murders of women are committed by intimate partners (WHO, 2013). Recent studies highlight a progressive increase in gender violence in intimate relationships, especially in adolescent population (Karakurt and Silver, 2013; Rodríguez and Megías, 2015; Taylor and Mumford, 2016). Considering these stunning data, dating violence is considered a human rights violation and public health issue throughout the world (Campbell, 2002; Garcia-Moreno et al., 2006). This fact leads us to the need of understanding what elements are contributing to the resistance and to the increase of dating violence.

In this mini-review, we propose an analysis of current studies on dating violence (for example, Fernández and Fuertes, 2010; Martsolf et al., 2012; Tapp and Moore, 2016), that focused the problem on percentages and statistics and on a woman centered perspective, often from a paternalistic approach. Later, we insert the study of gender models as a key element to shift attention from the study of the consequences or factors involved in gender violence to the analysis of the causes and of the structural elements that contribute to the reproduction of gender relations supported on a binary, complementary and excluding basis, starting from a gender relational perspective. To conclude, this perspective is proposed as innovative especially for its constant dialog with social movements and with the claims of collective rights and history of the different contexts, allowing the management and evaluation of gender policies in a more precise way to achieve equality between men and women.

## Dating Violence: Between Victimization, Paternalism and Percentages

Violence against women is a theme that draws the attention of state governments, international and global organizations for its pervasiveness, cultural transversality, and persistence. Gender violence is an urgent subject on the agenda, whose visibility has certainly been increased in recent years thanks to international pressure and to increasingly pervasive campaigns, allowing the observation of some of the main trends of gender studies and main gender policies. However, it has usually been perceived as a female problem. On the one hand, this fact sheds lights on the endless discrimination toward women, which involves them in a persistent struggle that constantly affects their lives. On the other hand, it strengthens a type of intervention that focuses more on the consequences than on the causes of the phenomenon, for example giving assistance to female victims of violence and trying to understand psychological, social and health consequences. There is a trend strongly built on helping women and the analysis of their condition as victims, which does not consider the relational structure in which women are inserted (Taylor and Mumford, 2016). In fact, a significant proportion of women victims of gender violence have experienced a long history of polyvictimization and revictimization (Fernández-González et al., 2017).

The need to make the phenomenon more visible, a particularly difficult operation for its main development in private space, has produced various studies that validate main international programs, which highlight the number of women victims of gender violence and dating violence (for example, WHO, 2013; European Union Agency for Fundamental Rights, 2014). These studies, fundamental for the understanding of the pervasiveness of the phenomenon, do not, however, allow a deeper analysis of the causes and of “gender order” (Connell, 1987). Gender violence is the top of the iceberg, the most extreme and definitive consequence of a patriarchal system that reproduces unequal gender relations, hierarchically ordered, complementary and excluding, based on a dual basis, masculinity and femininity gender models. This violence is instrumental, and its goal is to control and submit women who do not agree with patriarchal models.

## The Study of Gender Models: From Consequence to Cause Analysis of Dating Violence

Starting from the second feminist wave, and from the need, no longer postponed, to include women in the dominant power systems and to tackle a deep revision. We begin to focus our attention on how the patriarchal system creates structures of domination and subordination based on the existence of a binary reference system that is essential on the basis of sexual difference; this system is based on the model of masculinity and femininity. For gender models, we mean the set of characteristics, values, attitudes, roles, expectations that are expected of a person identified biologically as a man or a woman. This concept is also known as gender schema (Monreal Gimeno and Martínez Ferrer, 2010). The principal characteristics of the gender models as key elements in the reproduction of patriarchy:

(1) Gender models give us normative indications on how we must be in every aspect of our existence, making it easier for us to recognize Others and consequently, ourselves, in the society. They are not exclusively structures of social control, but also elements useful in defining our own identity. They, therefore, regulate objective aspects of life in society, as well as subjective aspects of the life of the individuals taking part in it.
(2) Gender models are binary, excluding but at the same time complementary. In the congruent model, masculinity and femininity are perceived as opposite extremes, excluding but complementary to each other (Santoro, 2018) even if hierarchically ordered. Opposition to femininity defines masculinity, while femininity can be understood as an absence, a lack of masculinity, in an immanent subordination. Main characteristics of masculinity and femininity models are presented in **Table 1**.

**Table 1:** Summary of masculinity and femininity models main characteristics (Santoro, 2018).

Masculinity	Femininity
Subject	Object
Three main refusals: not be child, not be woman, not love other men	Limit to masculinity
Dominant *Habitus*	Submissive *Habitus*
Impersonal *Habitus*	Expressiv*e Habitus*
Independence	Codependence
Unattainable ideal	Nature
Producer	Reproducer
Virility	Passivity
(3) Gender models are often invisible and reproducible. Being born from supposed biological bases and being socialized from the very early age through an education conforming to our gender reference models are not often perceived as a restriction on individual possibilities, but as a matter of fact, as the only and possible configuration of reality. Moreover, in this socialization phase, the process reproduces a specific gender model, in an exclusionary way.
(4) Gender models are dynamic. We are not defined monolithically by the gender socialization we receive: gender identity is created in a certain gender culture, which limits the possibilities of expression and self-realization, giving us a definite set of options. At the same time, gender identity depends on our personal experiences, in the form of education we receive, on contacts we have with other realities, on our ethical choices.

## Gender Relationships and Dating Violence: A New Perspective in Gender Studies

Integrating gender model analysis into the study of dating violence means shifting attention from a woman as a violence victim, to gender relations, taking into attention how and why this violence is the result of relationships of power socially and culturally built and reproduced. This perspective can only be integrated into the understanding of gender concept as relational and multidimensional. The internal differences of gender studies highlight the complexity of the gender concept and identify at least three, continually interconnected dimensions (Santoro, 2018). Gender is above all a personal identity variable, which allows the definition of others and myself. Gender also has a cultural, historical and social dimension. The structural dimension of gender, as a cultural system for organizing sexual difference. Moreover, gender is inserted into a power structure: in fact, its normative contents are constantly organized hierarchically, positively or negatively valuing, on the basis of binarism and heteronormativity (Tinat, 2016), that is shown in discrimination against no binary identity and sexual identities (Rollè and Marino, 2011; Ciocca et al., 2017).

Starting from a relational perspective, gender relations are compromise between social and cultural content, the dimension of power and personal gender identity. Including these elements in the study of dating violence means integrating a certain complexity that has two main consequences: analyze the phenomenon by going back to its possible causes, and. to make clear how gender violence, in all its forms and manifestations, is the most extreme consequence of a complementary, exclusionary and unequal relations system, which feeds on gender-normative content and also contributes to its own identity definition. Despite these historical changes, the binary reference structure of gender models of masculinity and femininity remains in force, causing movements, dynamics and contradictions that can be related to dating violence.

Firstly, there is a greater resistance of gender models by men than by women. In fact, if the model of femininity has been the subject of a profound revision process, which has led to important changes in the lives of women, that of masculinity has remained fixed. Secondly, there is a constant contradiction between practices and beliefs related to gender models. Despite women’s integration in both education and in the workplace the normative models which constitute the sphere of ideas and beliefs continue to be the gender material which constitutes and regulates the society in which we live. As Monreal Gimeno and Martínez Ferrer (2010) states, gender patterns maintain certain autonomy despite the real changes in the characteristics associated with this group.

Starting from these considerations, and from the results of recent research on gender relations and dating violence, especially in adolescents, it is clear how these elements are related. The freedom of women, which extends beyond the model of femininity that sees women as mother, wife, passive, sensitive, docile and attentive to others, puts in crisis the same identity recognition of man, with whom it relates, which becomes an expression of the norm that re-establishes the predetermined order. Moreover, the same binary power system is based on excluding opposites, incommunicable, but at the same time complementary. At the same time, this difference is justified by the myth of romantic love or the “soul mate”. In this discourse, man and woman, however different, are complementary and need this union to reach fullness (Cubells and Calsamiglia, 2015).

## Conclusion and Future Directions

The perpetuation of violent relationships that have seen women victims of every kind of abuse, deprivation, humiliation and physical and psychological damage, over the centuries goes hand in hand with a certain gender training that is put in place in order to reproduce an order of established gender hierarchy, which relies on models of masculinity and femininity that self-confirm and justify each other. For this reason, the dialog between studies on gender models with those on dating violence, allows us to reconstruct the network of gender relations in which we are constantly immersed to understand the tendencies, contradictions, difficulties that are at the base of the phenomenon of gender violence. Furthermore, this new understanding allows to have the elements for a direct intervention on differential socialization process, that transmit different gender values for men and women (McCarry, 2010; Santoro, 2018). In fact, gender violence is not characterized by its exceptionality, but by its complexity: considering gender as a multidimensional and relational variable, it is possible to understand what the causes of this phenomenon are, provoking an important change of perspective with respect to the development of policies aimed at preventing gender-based violence. For this reason, gender equality intervention should be started in early age, and directed to boys and girls, working on deconstruction of gender roles and gender attributes, striking gender as a vehicle of personal and continuous identity construction, a chance to express yourself starting from your own strengths and debilities, not limited by models or role. Working on gender models means a continuous process in which education institutions could strike a central role (Biemmi, 2015), giving the opportunity to find a right and comfort space to debate, to experience, to express and create an equality culture and values. Especially, focusing on emotional education (Leathwood and Hey, 2009). and spreading dialog on real and daily discrimination cases and on instruments that allow permanent stereotypes reproduction, as mass media and social network (Tortajada, 2013).

These policies must consider the need to put in tension, to open spaces of possibilities, to eliminate the normativity of gender models that continue to be static and to influence people’s lives. Similarly, and starting from a perspective that includes gender as relational, it seems fundamental to exit from a vision that binds gender violence as a “feminine” problem: the construction of unequal relationships originates from a binary basis, which sees equally involved men and women. For consequence, it’s fundamental to integrate men as a direct actor and beneficiary of gender policies. It has been pointed out that there are cultural differences in aggressors in aspects related to gender models such as the acceptability of violence against women in relationships, sexist beliefs about male domination and honor, and the role of women as caregivers of the family (Lila et al., 2013; Vargas et al., 2015). At the same time, integrating the understanding of these elements to the study of dating violence means working on the development and implementation of efficient and at the same time complex measuring instruments; research should not be focused only on percentage and statistics on gender violence’s victims and perpetrators, but also on the diffusion of an equality culture and, on the opposite, on gender models’ resistance. Focus on ideas and beliefs states a change from consequences to a causes approach to gender violence, and, for instance, from restraint to prevention policies. In general, it means looking at complexity in gender studies, which increasingly embraces an approach to transversality, relationality, intersectionality, and which can, therefore, be the basis for the development of gender policies that are truly effective with respect to the integral goal of equality between men and women. Today, we can develop integral equality plan, directed to ends with gender discrimination, only taking into account and knowing our context’s gender culture resistances and changes, helping men and women to reflect and reconstruct their own relationships starting from their own needs and opportunities, instead of on their own fear to not be social recognized as “good,” “complete,” “proper” man and woman.

## Author Contributions

CS, BM-F, CM, and GM had participated in the intellectual content, the analysis of data, and the writing of the work. CS, BM-F, CM, and GM had reviewed the final version of the work and they approve it for publication.

## Conflict of Interest Statement

The authors declare that the research was conducted in the absence of any commercial or financial relationships that could be construed as a potential conflict of interest.
